# Supplementary material for: Constructing a Glioblastoma Prognostic Model Related to Fatty Acid Metabolism Using Machine Learning and Identifying F13A1 as a Potential Target
Source: Biomedicines. 2025 Jan 21;13(2):256. doi: 10.3390/biomedicines13020256 (PMC11852379; doi:10.3390/biomedicines13020256)
Supplement: Supplementary file 1 [file biomedicines-13-00256-s001.zip › Signature generated from machine learning based integrative approaches.pdf]

Signature generated from machine learning based integrative approaches

To develop a construct prognostic model with high accuracy and stability performance, we integrated 10 machine learning algorithms including random survival forest (RSF),Lasso,generalized boosted regression modeling (GBM),CoxBoost,stepwise Cox,partial least squares regression for Cox (plsRcox),supervised principal components (SuperPC),Ridge,elastic network (Enet) and survival support vector machine (survival-SVM). We used 101 combination algorithms to construct prognostic models on training set(TCGA-GBM) and testing sets(GSE43378 and GSE83300) based on the leave-one-out cross-validation (LOOCV) framework.

Here are the details of the 10 models:

Model 1: The RSF model was implemented via the randomForestSRC package. RSF had two parameters ntree and mtry, where ntree represented the number of trees in the forest and mtry was the number of randomly selected variables for splitting at each node. We used a grid-search on ntree and mtry using LOOCV framework. All the pairs of (ntree, mtry) are formed and the one with the best C-index value is identified as the optimized parameters. The formula of the model is as follows:

$$S(t|X) = \frac{1}{B}\sum S_k(t|X)$$

S(t|X) is the survival function at time t given the covariates X.

S_k(t|X) is the survival function given by the kth tree.

B is the number of decision trees.

Model 2: The Lasso was implemented via the glmnet package. The regularization parameter, λ, was determined by LOOCV. The formula of the model is as follows:

$$\text{Cost}(w) = \sum_{i=1}^{N} (y_i - w^T x_i)^2 + \lambda\|w\|_1$$

w is the vector of weight coefficients.

x_i is the feature vector for the i-th sample.

y_i is the target value for the i-th sample.

N is the number of samples.

λ is the penalty coefficient, controlling the strength of regularization.

$||w||_1$ is the L1 norm of w, which is the sum of the absolute values of each element in w.

Model 3: The Enet was implemented via the glmnet package and α was set to 0-1 (interval =0.1).Enet mainly consists of an encoder and a decoder. Its core idea is to achieve pixel-level classification through multi-level feature extraction and upsampling operations. In the encoding stage, the network extracts image features through layer-by-layer convolution and pooling operations. In the decoding stage, the network restores the extracted features to the size of the original image through upsampling and fusion operations, and outputs the classification result for each pixel.

Model 4: The Ridge were implemented via the glmnet package and λ was determined by LOOCV.The formula of the model is as follows:

The calculation formula for the regression coefficient β:

$$\beta = (X^T X + \lambda I)^{-1} X^T y$$

X is the design matrix, which contains data on the independent variable features.

X^T is the transpose of the design matrix.

y is a vector of response variables and target variables.

I is the identity matrix.

λ is the shrinkage penalty term, also known as the regularization parameter, which is used to control the size and complexity of the model and prevent overfitting.

The cost function:

$$J(\beta) = \sum(y - X\beta)^2 + \lambda\beta^2$$

$\sum(y-X\beta)^2$ is the sum of squared residuals.

$\lambda\beta^2$ is a regularization term used to penalize the regression coefficients

Model 5: The GBM model was implemented via superpc package. Using the LOOCV, the cv.gbm function selected index for number trees with minimum cross-validation error. The gbm function was used to fit the generalized boosted regression model. The basic principle of GBM is to improve previous models by iteratively adding new models, gradually improving the accuracy of prediction. In each iteration, GBM tries to reduce the prediction error, i.e. the loss function. To achieve this goal, GBM uses gradient descent to minimize the loss function.

Model 6: The CoxBoost model was implemented via CoxBoost package, which is used to fit a Cox proportional hazards model by componentwise likelihood-based boosting. For the CoxBoost model, we used LOOCV routine optimCoxBoostPenalty function to first determine the optimal penalty (amount of shrinkage). Once this parameter was determined, the other tuning parameter of the algorithm, namely, the number of boosting steps to perform, was selected via the function cv.CoxBoost. The dimension of the selected multivariate Cox model was finally set by the principal routine CoxBoost.The working principle of the CoxBoost model is based on the enhancement of component likelihood to fit the Cox proportional hazards model, and uses an enhancement method based on offset.

Model 7: The stepwise Cox model was implemented via survival package. A stepwise algorithm using the AIC (Akaike information criterion) was applied, and the direction mode of stepwise search was set to "both", "backward", and "forward", respectively. The stepwise Cox model does not possess a specific mathematical formula, as it is a stepwise regression process used for selecting variables in the Cox proportional hazards model. This process encompasses methods such as forward selection, backward elimination, and bidirectional elimination, to gradually construct the optimal Cox proportional hazards model.The basic formula of Cox proportional hazards model is as follows:

$$h(t, X) = h0(t) * \exp(\beta'X)$$

h(t, X) represents the hazard function at time t and with covariates X.

h0(t) is the baseline hazard function, related to time t but independent of covariates X.

$\beta'X$ is the linear combination of covariates X, where β is the vector of regression coefficients and X is the vector of covariates.

Model 8: The plsRcox model was implemented via plsRcox package. The cv.plsRcox function was used to determine the number of components requested, and the plsRcox function was applied to fit a partial least squares regression generalized linear model. The plsRcox model is an algorithm based on partial least squares regression (PLS) and Cox regression, used for survival analysis of high-dimensional data.

Model 9: The SuperPC model was implemented via superpc package, is a generalization of principal component analysis, which generates a linear combination of the features or variables of interest that capture the directions of largest variation in a dataset. The superpc.cv function used a form of LOOCV to estimate the optimal feature threshold in supervised principal components. To avoid problems with fitting Cox models to small validation datasets, it uses the "pre-validation" approach. The SuperPC model is an algorithm that considers survival time during dimensionality reduction. Its core idea is to perform principal component analysis on genes closely related to survival time.

Model 10: The survival-SVM model was implemented via survivalsvm package. The regression approach takes censoring into account when formulating the inequality constraints of the support vector problem.The survival-SVM model is a support vector machine (SVM) method used for survival analysis. It combines the powerful classification ability of SVM with the characteristics of survival analysis, and can handle problems involving time-event data such as survival time and survival status.
