# Supplementary figures and images for: Constructing a Glioblastoma Prognostic Model Related to Fatty Acid Metabolism Using Machine Learning and Identifying F13A1 as a Potential Target
Source: Biomedicines. 2025 Jan 21;13(2):256. doi: 10.3390/biomedicines13020256 (PMC11852379; doi:10.3390/biomedicines13020256)

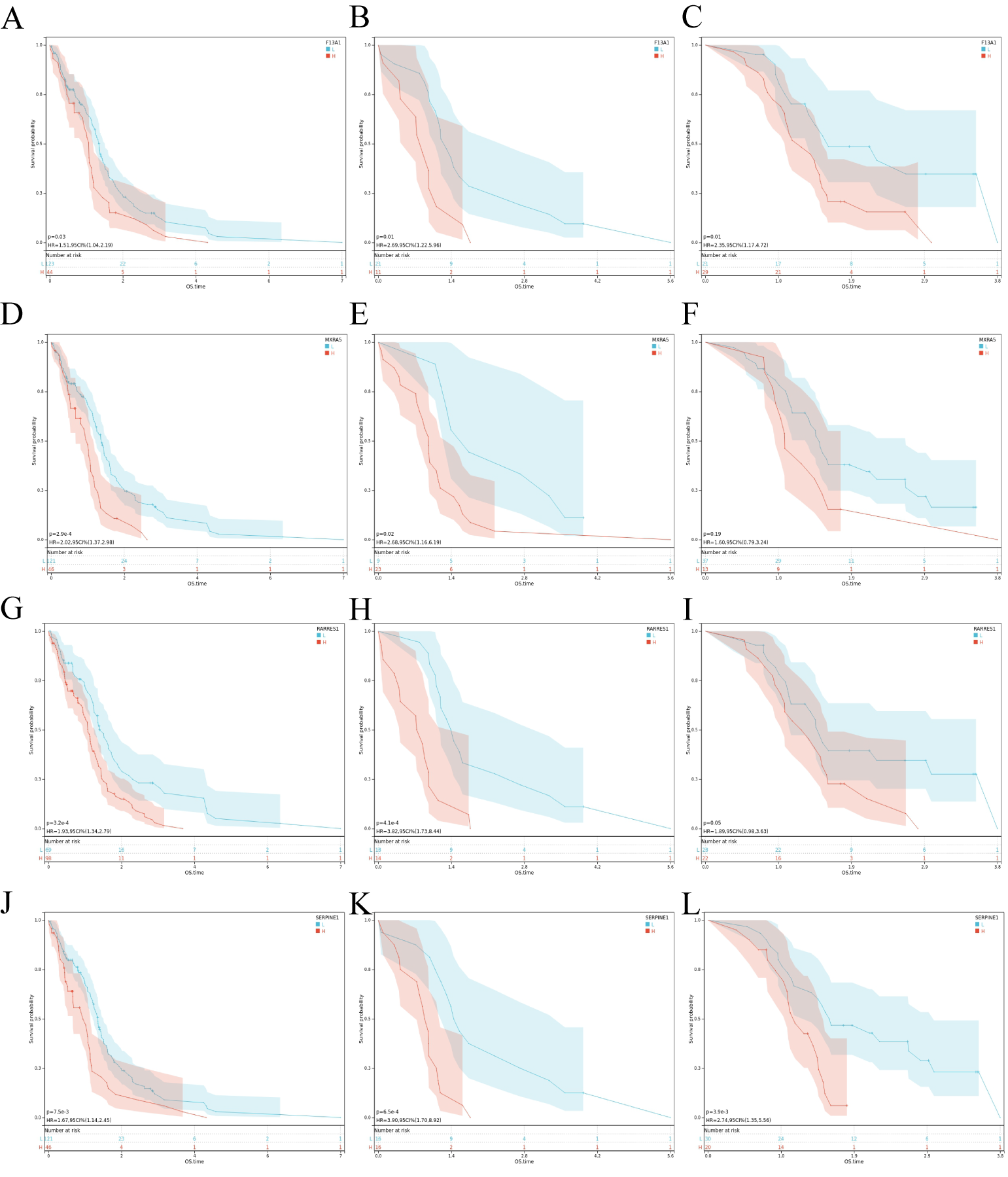

Supplement: Supplementary file 1 [file biomedicines-13-00256-s001.zip › Figure S1.tif]

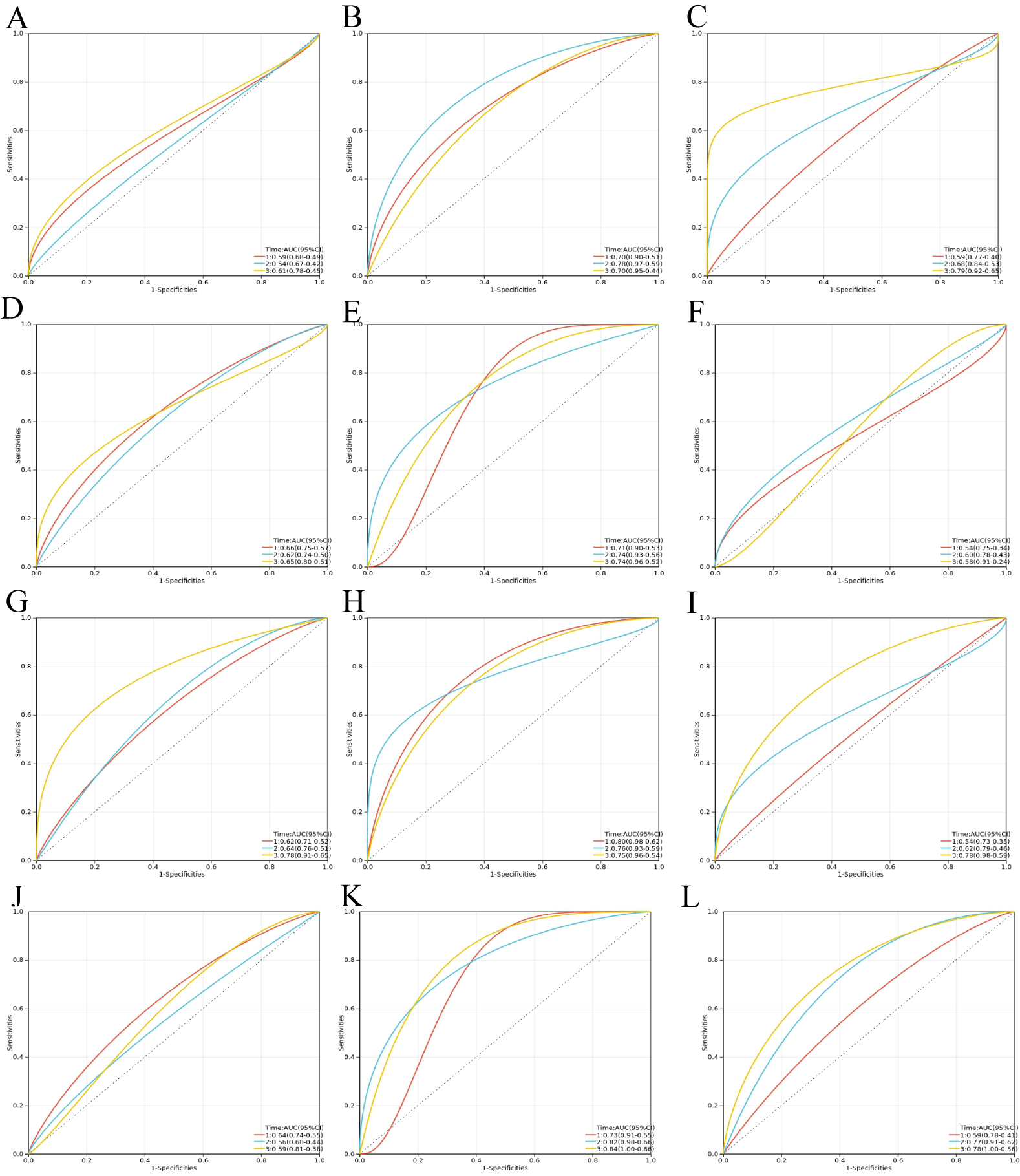

Supplement: Supplementary file 1 [file biomedicines-13-00256-s001.zip › Figure S2.tif]

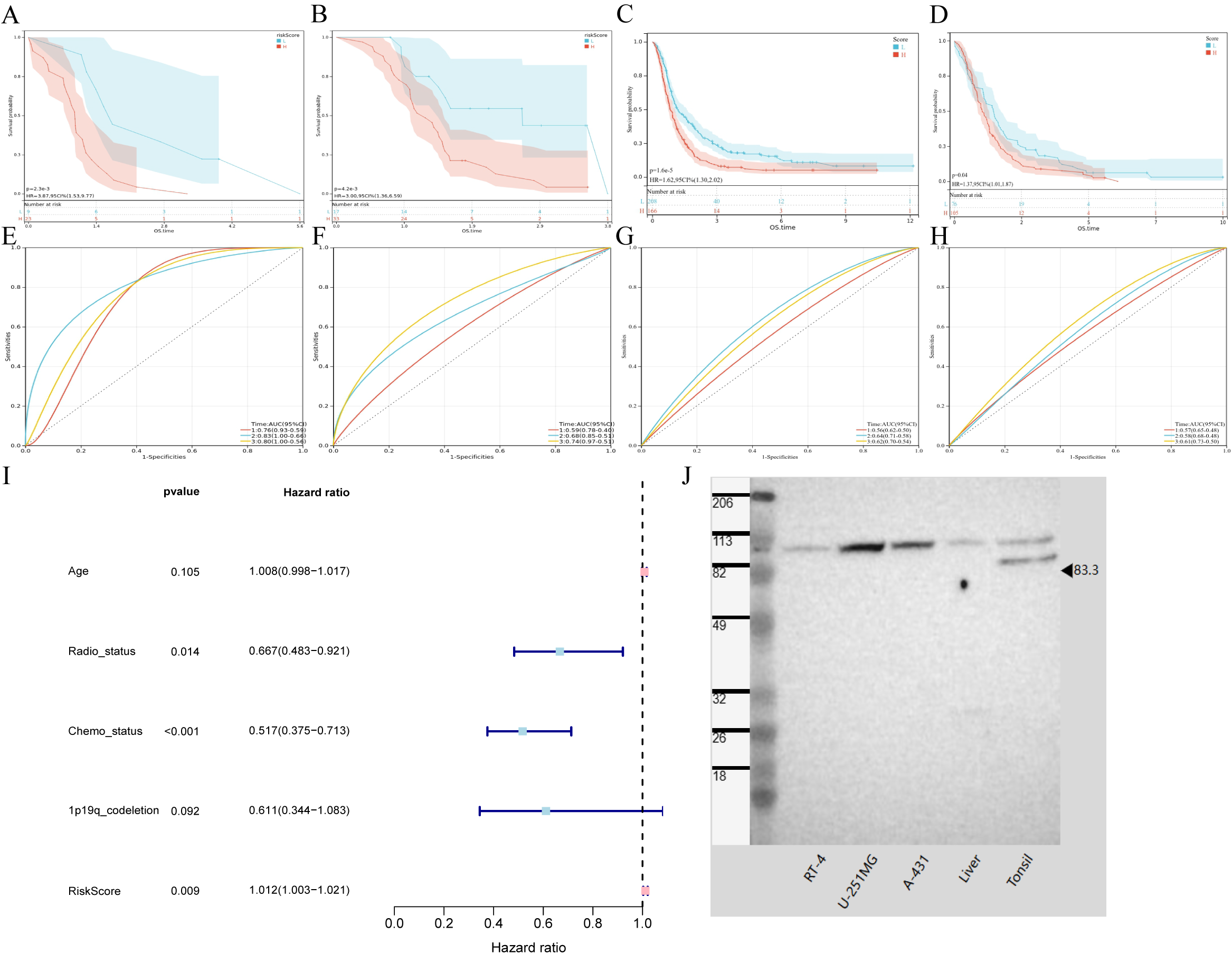

Supplement: Supplementary file 1 [file biomedicines-13-00256-s001.zip › Figure S3.tif]
